# Anion exchange in inorganic perovskite nanocrystal polymer composites†
†Electronic supplementary information (ESI) available: Experimental details on the synthesis of the NCs, the preparation of the polymer:NCs nanocomposites and the anion exchange processes as well as optical and structural characterization of the nanocrystals and the nanocomposites are presented. See DOI: 10.1039/c8sc02830c


**DOI:** 10.1039/c8sc02830c

**Published:** 2018-08-30

**Authors:** Maria Sygletou, Maria-Eleni Kyriazi, Antonios G. Kanaras, Emmanuel Stratakis

**Affiliations:** a Institute of Electronic Structure and Laser , Foundation for Research and Technology – Hellas , Heraklion , 71110 , Crete , Greece . Email: masyg@iesl.forth.gr ; Email: stratak@iesl.forth.gr; b Physics and Astronomy , Faculty of Physical Sciences and Engineering , University of Southampton , Southampton , SO171BJ , UK; c Department of Materials Science and Technology , University of Crete , Heraklion 71003 , Crete , Greece

## Abstract

We demonstrate a facile, low-cost and room-temperature method of anion exchange in cesium lead bromide nanocrystals (CsPbBr_3_ NCs), embedded into a polymer matrix. The anion exchange occurs upon exposure of the solid CsPbBr_3_ NCs/PDMS nanocomposite to a controlled anion precursor gas atmosphere. The rate and extent of the anion exchange reaction can be controlled *via* the variation of either the exposure time or the relative concentration of the anion precursor gas. Post-synthesis chemical transformation of perovskite nanocrystal–polymer composites is not readily achievable using conventional methods of anion exchange, which renders the gas-assisted strategy extremely useful. We envisage that this work will enable the development of solid-state perovskite NC optoelectronic devices.

## Abstract 2

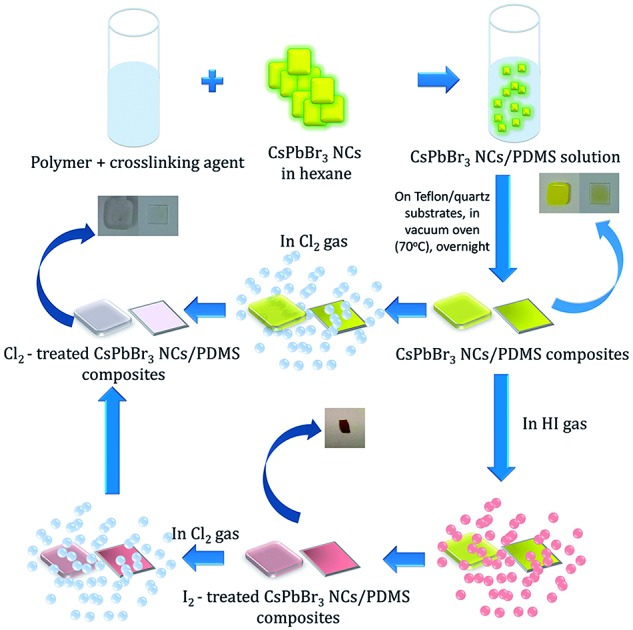
We demonstrate a facile, low-cost and room-temperature method of anion exchange in cesium lead bromide nanocrystals (CsPbBr_3_ NCs), embedded into a polymer matrix.

## 

Solution-processed all-inorganic cesium lead halide perovskite (CsPbX_3_, X = Cl, Br, I) nanocrystals (NCs) have drawn a lot of attention lately, due to their exceptional optical properties, including medium optical bandgaps, strong absorption coefficients, high luminescence quantum yields and narrow emission bandwidths.^1^–^5^ Owing to these properties, they have been introduced as a new class of photoactive materials for next-generation, low-cost, high-performance flexible optoelectronics,^6^,^7^ including perovskite-based solar cells,^8^ lasing sources,^9^,^10^ photodetectors^11^ and light-emitting diodes^12^–^15^ with high brightness and tunable emission. At the same time, all-inorganic perovskites exhibit higher thermal and chemical stability,^16^ as well as higher resistance to humidity^17^ than their organic–inorganic counterparts, such as MAPbX_3_. The stability of halide perovskite NCs still remains a research topic of great interest.^18^ It has been reported that the robustness of CsPbX_3_ NCs can be improved by the addition of a small amount of polymer (poly(maleic anhydride-*alt*-1-octadecene)-PMA into the precursor solutions, which creates an additional ligand coating around each individual NC, or *via* encapsulation into PMMA or polyethylene oxide).^12^,^19^–^21^ Furthermore, a silica-coating process has been reported to enhance the stability of inorganic perovskite NC-based LEDs.^22^,^23^ A prominent property of perovskite NCs is their ability to undergo a post-synthesis anion exchange, in solution, using chemical precursors or photo-induced processes.^24^–^27^ Despite the numerous studies on anion exchange reactions in the liquid phase, only a few reports have demonstrated such reactions in solid state, either in the bulk or in the form of NCs. In particular, Hoffman *et al.*^28^ reported the conversion of CsPbBr_3_ to CsPbI_3_ films following heat treatment with a PbI_2_ solution. While, Guhrenz *et al.*^27^,^29^ reported a method of anion exchange *via* the direct incorporation of CsPbX_3_ NCs into ion-rich matrices. In parallel, there have been reports of post-synthetic halide exchange reactions in organic–inorganic metal-halide bulk perovskites (OIHPS) upon exposure to halogen (X_2_)^30^–^32^ and hydrogen halide (HX) gases.^33^ Gas-induced formation/transformation (GIFT) of OIHPS has shown tremendous promise in various applications, including solar cells, optoelectronics, sensors, and beyond, however, a detailed understanding of the mechanisms underlying the GIFT phenomena is still lacking.^31^

In this communication, we introduce for the first time a GIFT process in perovskite NCs in solid state. In particular, we present a simple, post-synthesis and room temperature, solid-state anion exchange method to tune the emission properties of inorganic perovskite NCs, hosted into a polymer matrix. We demonstrate anion exchange in nanocomposite layers, comprising of CsPbBr_3_ NCs dispersed in polydimethylsiloxane (PDMS), upon their exposure to a halide precursor gas atmosphere at room temperature. Fig. 1 represents a schematic illustration of the schematic route followed for the transformation of CsPbBr_3_ to CsPbCl_3_. It is shown that the extent of the anion exchange reaction and therefore the NCs' emission properties can be finely tuned by adjusting the exposure time and concentration of Cl_2_ gas; the iodine anion exchange process is also demonstrated. Apart from the tunability of nanoparticle emission, it is shown that the PDMS matrix protects the NCs against adverse humidity effects, giving rise to stable optical properties. These properties can open up new avenues for the *in situ* and low-cost optical modulation of perovskite polymer-nanocomposites, useful in various optoelectronic applications.

**Fig. 1:**
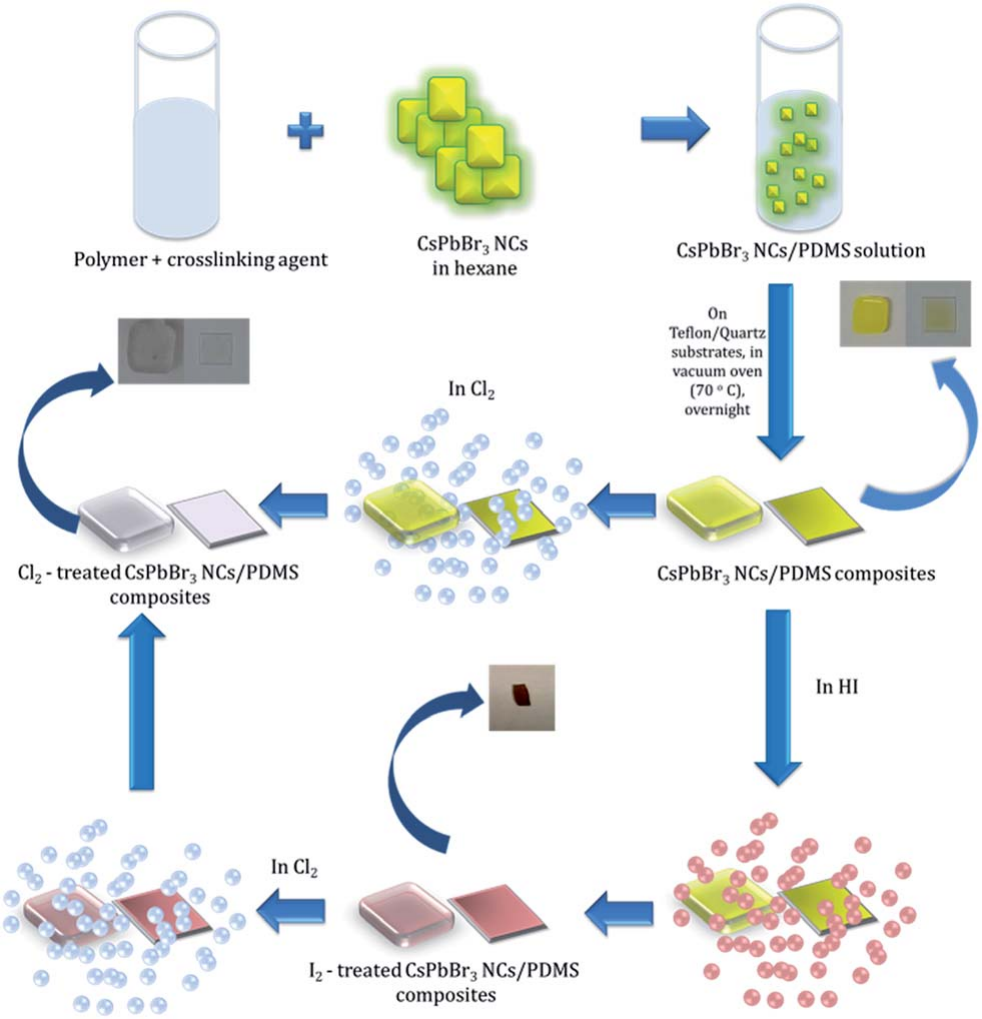
Process flow of post-synthesis anion exchange in CsPbBr_3_ NCs in solid phase due to Cl_2_ and/or HI treatment.

Following synthesis, the NC colloids in hexane showed a characteristic fluorescence peak at 521 nm, with a full width half maximum (FWHM) of ∼25 nm (Fig. S2†). The incorporation of NCs into PDMS^34^ gave rise to a nanocomposite with a characteristic yellowish color under ambient light (Fig. 2, inset) and a pronounced green emission upon excitation with UV light (Fig. 3b). As shown in Fig. 2, the NCs' absorption maximum was slightly red-shifted from 495 nm in solution to 510 nm in the nanocomposite, while the fluorescence maximum was slightly blue-shifted from 521 nm in solution to 515 nm in the nanocomposite (Fig. 2). This is mainly due to the increase in the dielectric properties of the surrounding medium, from hexane with *n*_hexane_ = 2.06, to PDMS with *n*_PDMS_ = 2.3–2.8. Furthermore, a slight broadening of the respective emission peak was observed due to the formation of NC clusters, which was by Two-Photon Excited Fluorescence (TPEF) Microscopy (Fig. S4†).

**Fig. 2:**
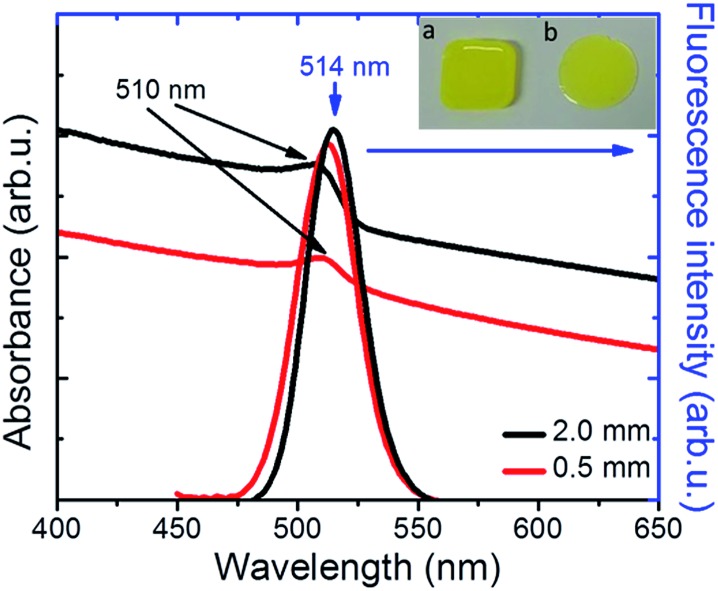
Normalized UV-Vis absorption and fluorescence spectra of PDMS:NCs nanocomposites. The inset shows pictures of nanocomposites (a) formed on a Teflon mould (thickness of 2 mm) and (b) drop-casted onto a quartz substrate (thickness of 0.5 mm).

**Fig. 3:**
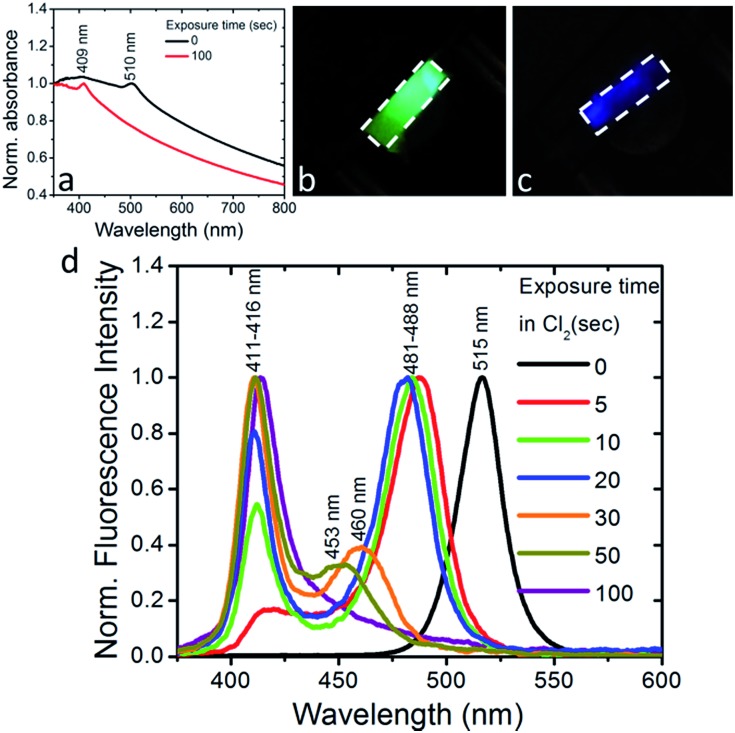
(a) Normalized UV-Vis absorption spectra of PDMS:CsPbBr_3_ NC nanocomposite layers of 0.5 mm thickness, before and after exposure to Cl_2_ gas with a partial pressure of 70 mbar for 100 s. Images of a PDMS:CsPbBr_3_ NC composite layer upon UV excitation, before (b) and after (c) exposure to Cl_2_ gas. The sample area is marked with the white dashed line. (d) Normalized fluorescence spectra of PDMS:CsPbBr_3_ NC nanocomposite layers of thickness 0.5 mm, before and after exposure to Cl_2_ gas for various time intervals.

More importantly, the emission of NCs hosted into PDMS was observed to be remarkably stable over time, upon exposure to ambient conditions. This is in contrast to the widely reported sensitivity of CsPbBr_3_ NCs to ambient air and/or moisture.^35^–^37^ To further explore such emission stability and robustness against humidity, we investigated the emission spectra evolution of the PDMS:CsPbBr_3_ NC layers, following their immersion into water. It was observed that the prolonged (24 h) interaction of the nanocomposites with water caused no significant effect on their respective emission spectra (Fig. S5†). Furthermore the fluorescence spectra of the nanocomposites remain practically unchanged upon storage of the nanocomposites for 30 days in ambient conditions (Fig. S5†). Both of the above observations are strong indications that the polymer matrix successfully protects the NCs against the effects of humidity.

We also observed that the optical absorption and fluorescence spectra of the PDMS:CsPbBr_3_ NC layers progressively blue-shifted upon their exposure to Cl_2_ gas, indicating the anionic exchange of the participating halides. The solid-state chlorination process is presented in the ESI.† Representative results are shown in Fig. 3. In particular, exposure to Cl_2_ gas, of 70 mbar-partial pressure, for 100 s gave rise to a blue-shift of both the absorption and emission peaks from ∼510 nm to ∼410 nm. This shift is reasonable, considering that the emission peak of CsPbBr_3_ NCs is around 510 nm while that of CsPbCl_3_ NCs is observed at ∼390 nm.^24^ At the same time, quenching of the fluorescence quantum yield was observed. Both phenomena, *i.e.* the partial replacement of Br ions with Cl ions and the fluorescence quenching are in accordance with former findings^24^,^25^ regarding NC colloids. It should be noted that the FWHM of the blue-emitting composite layers attained is comparable to that of the initial layers. In addition, an incomplete exchange reaction took place for the thickest (∼2 mm) samples tested. This is presented in Fig. S6,† showing that two characteristic absorption peaks, at ∼409 nm and ∼465 nm, arise upon exposure of the sample to a chlorine environment (Fig. S6†). The corresponding fluorescence spectra confirm the emission from two peaks, at ∼411 nm and ∼475 nm, with the latter being the most pronounced (Fig. S7†). This is possibly due to the formation of mixed halide CsPb(Br/Cl) NCs with different Cl : Br ratios. On the contrary, in the case of a thinner layer (∼500 μm), a single absorption peak at 409 nm is observed (Fig. 3a), while at the same time the emission peak shifts from 515 nm (Fig. 3d, black line) to 411 nm within 100 s of exposure to chlorine (Fig. 3d, violet line), indicating the formation of CsPb(Br/Cl) NCs with a Br : Cl ratio of 2 : 3.^24^ Following the exposure for 100 s, the phenomenon is partially reversible (Fig. S15†), *i.e.* the fluorescence spectrum slowly red-shifts with time and saturates to a peak emission value of 475 nm, attributed to the chemical composition of CsPbBr_3_Cl_2_ NCs (Br : Cl ratio of 3 : 2). In Fig. 3b and c typical images of a nanocomposite layer under UV light excitation, before and after exposure to Cl_2_, are presented, respectively. It can be clearly seen that, the emitted green color of the pristine sample changes to blue upon chlorine treatment. Also, as shown in Fig. S8,† the color of the respective sample changes from yellow to light grey. In literature, anion conversion reactions have already been interpreted in terms of halogen reduction potentials, at least in the case of OIHPs.^30^ These studies showed that exposure of OIHPs to a halogen gas, X_2_, can displace the crystal halide anions, Y^–^, at room temperature, provided that X features a higher standard reduction potential than the displaced halide, Y.^30^,^31^ Our results indicate that this could also occur in the all-inorganic lead halide perovskites as well. Considering the higher reduction potential of Cl_2_ compared to Br_2_, Cl_2_ can oxidize Br^–^ and convert CsPbBr_3_ to CsPbCl_3_ with solely gas-phase by-products. In the case of PDMS:CsPbBr_3_ NCs, this process is facilitated by the high permeability and diffusivity of Cl_2_ gas in PDMS,^38^ enabling chlorine atoms to interact with the embedded perovskite NCs. Based also on the relevant literature, the flow rate of Cl_2_ gas across a PDMS membrane is proportional to the difference in partial pressure and inversely proportional to the membrane thickness;^39^ this could account for the deficient anion exchange process taking place in the thicker nanocomposite layers.

To further shed light on the anion exchange process, the exposure of the nanocomposite layers to different Cl_2_ gas partial pressures was investigated. The corresponding results are presented in Fig. 4 and S9;† in these figures *I*_1_ is the intensity of the initial emission peak (∼515 nm) and *I*_2_ is the intensity of the emission peak that emerges upon exposure to Cl_2_ (*i.e.* at ∼435 nm). It can be observed (Fig. 4a) that, as the Cl_2_ gas pressure is increased from 0 to 70 mbar, the initial emission peak progressively blue-shifts and *I*_1_ decreases, while, on the other hand, *I*_2_ gradually increases. It is also shown in Fig. 4b and c that both the *I*_2_/*I*_1_ intensity ratio and the 1^st^ emission peak shift tend to saturate at a similar Cl_2_ gas critical partial pressure (∼20 mbar). These observations indicate the potential of the perovskite nanocomposite layers to operate as halide gas sensing elements. It is notable that the fluorescence signal of these nanocomposites is preserved, even after 24 h of treatment with chlorine.

**Fig. 4:**
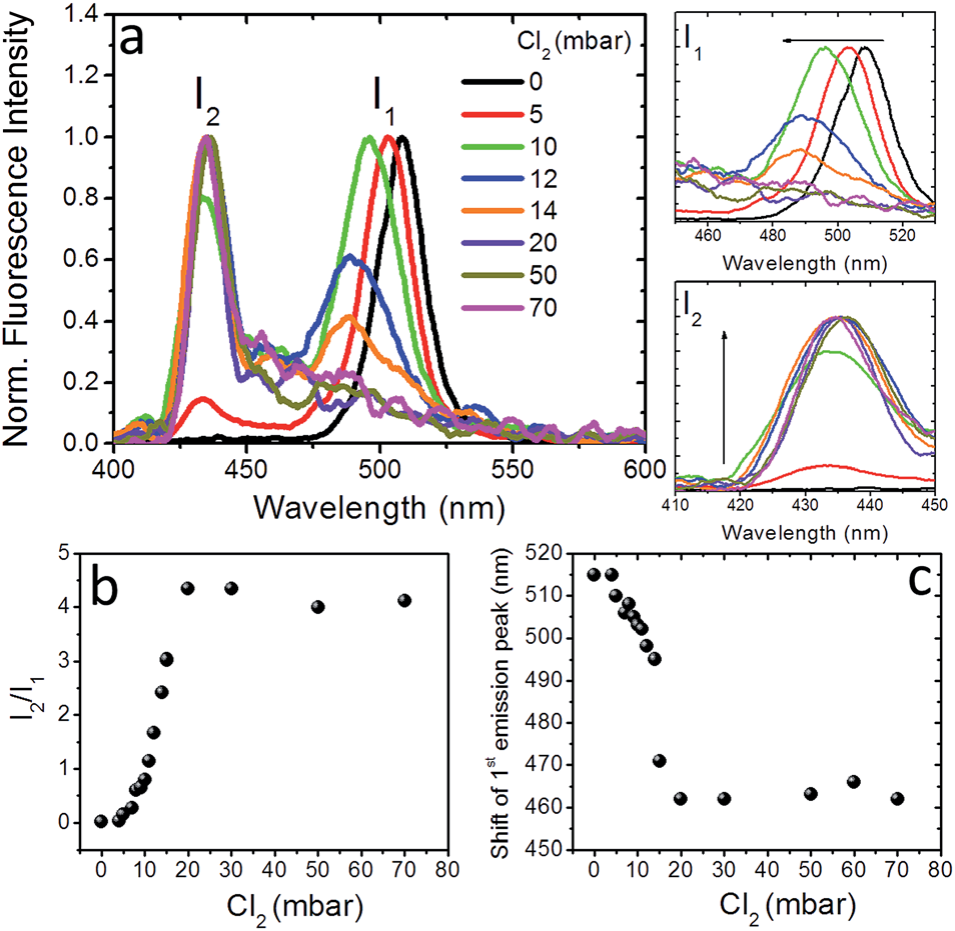
(a) Normalized fluorescence spectra of, 0.5 mm thick, PDMS:CsPbBr_3_ NCs nanocomposite layers upon exposure to different partial Cl_2_ pressures. The corresponding evolution of the initial peak with intensity *I*_1_ (top) and of the peak that emerges after chlorine treatment with intensity *I*_2_, (bottom) are shown on the right. (b) Fluorescence intensity ratio, *I*_2_/*I*_1_, and (c) spectral shift of the first emission peak, as a function of the partial pressure of Cl_2_ gas.

The photoluminescence quantum yield (PLQY) of the initial CsPbBr_3_ nanocrystals in hexane, measured *via* the comparative method,^40^ was equal to 48%. Compared to the nanocrystals in solution, it is observed that when an equal vol% of CsPbBr_3_ nanocrystals is embedded into PDMS, the photoluminescence intensity decreases (Fig. S16†). Accordingly, the corresponding PLQY measured for the PDMS:CsPbBr_3_ NC layers was dropped to 36%. Following chlorine treatment, the PLQY of the nanocomposites was decreased by almost 10 times, *i.e.* to 4%, which is in accordance to previous reports on the anion exchange effect on the PLQY.^24^

Experiments in the presence of an iodine precursor gas were also performed,^32^ as schematically shown in Fig. 1. The solid-state iodination process is presented in the ESI.† Fig. 5a and b present the absorption and fluorescence spectra of the PDMS:CsPbBr_3_ NC nanocomposite layers following sequential treatment, first with I_2_ gas under ambient conditions, followed by Cl_2_ gas. Following exposure to I_2_ gas under ambient conditions for 10 minutes, the nanocomposites showed a red-shifted emission peak at ∼660 nm (∼1.87 eV) that complies with that reported for CsPbI_3_ NCs.^24^ Subsequently, these nanocomposites were placed in a chlorine environment and their emission peak was observed to blue-shift to ∼410 nm (3.02 eV), *i.e.* close to that observed upon direct chlorination of the pristine PDMS:CsPbBr_3_ NC layers. Considering the lower reduction potential of I_2_ compared to that of Cl_2_, a redox-type conversion reaction, *i.e.* oxidation of Br^–^ by I_2_ and subsequent conversion of CsPbBr_3_ to CsPbI_3_, could not account for the observed displacement of the emission peak. However, it is well known that the ambient humidity remarkably affects the I_2_ gas stability, leading to the formation of HI and HIO.^41^ It has also been reported that mutual anion conversions in perovskite NCs can be alternatively realised upon exposure to gaseous HX, *via* ion-exchange reactions.^33^ Based on this, the possibility of HI formation due to ambient humidity may account for the observed red shift in the UV-Vis and fluorescence spectra. Experiments involving exposure of PDMS:CsPbBr_3_ NC nanocomposites to HX gases are currently in progress to clarify this issue.

**Fig. 5:**
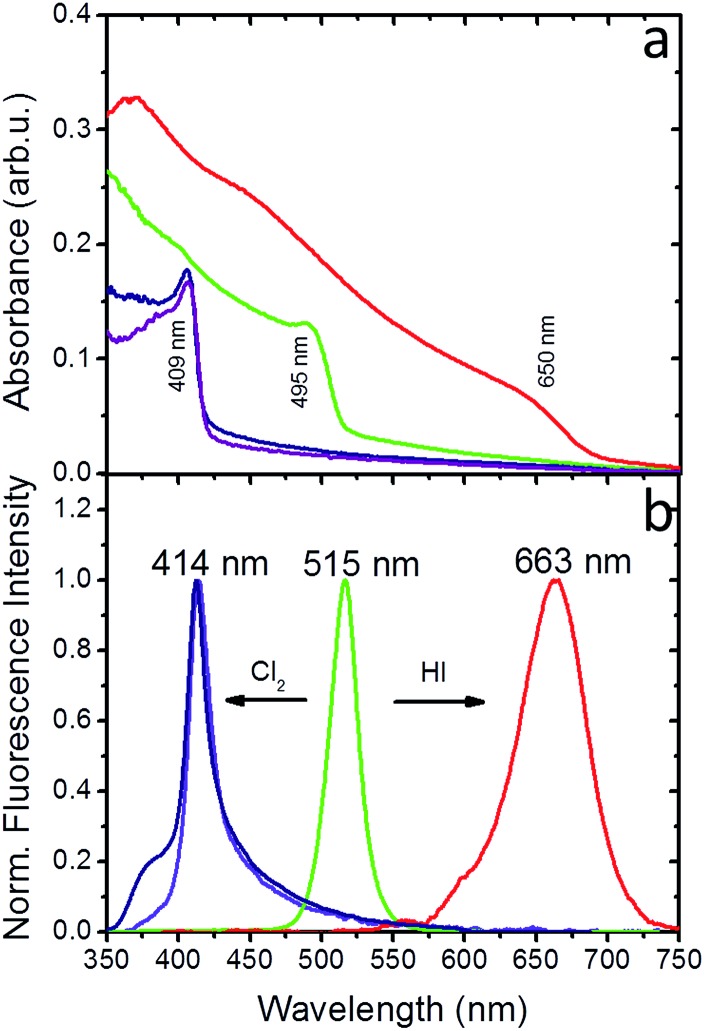
(a) UV-Vis absorption of PDMS:CsPbBr_3_ NC nanocomposite layer (green line), following Cl_2_ (purple line) and HI (red line) treatment for 10 minutes, as well as Cl_2_ treatment of the iodinated nanocomposite for 10 minutes (blue line). (b) Normalized fluorescence intensity of PDMS:CsPbBr_3_ NC nanocomposite layer (green line) following Cl_2_ (purple line) and HI (red line) treatment for 10 minutes, as well as Cl_2_ treatment of the iodinated nanocomposite for 10 minutes (blue line).

It can be concluded that the anion exchange process can only proceed along a single direction, that is Br^–^ > Cl^–^, Br^–^ > I^–^, I^–^ > Cl^–^. This is further confirmed by experiments with PDMS:CsPbI_3_ NCs nanocomposite layers showing a characteristic shift of the initial fluorescence peak to lower wavelengths upon exposure to Cl_2_ gas (Fig. S17 and S18†). Our findings comply with the reduction potential relationship of the three, considering that Cl_2_ exhibits higher reduction potential compared to Br_2_ and I_2_ exhibits higher reduction potential compared to Br_2_.

To further account for the microscopic mechanism behind the anion exchange process, FTIR, XPS and XRD spectra of the PDMS:CsPbBr_3_ NC layers, prior and after chlorine treatment, were recorded. The corresponding FTIR spectra, presented in Fig. S10 and S11,† reveal no significant change in the chemical structure of the nanocomposites following halogen gas treatment. The survey XPS scans (Fig. S12†), recorded from the samples before and after chlorine treatment, show mainly the presence of O, C and Si, attributed to the PDMS matrix.

Fig. S13† shows the respective high-resolution XPS spectra of Cs 3d, Pb 4f and Br 3d peaks. Prior to Cl_2_ exposure, traces of Cs, Pb and a small amount of Br were detected. While, after exposure to Cl_2_, traces of Cs, Pb and a small amount of Cl were detected, indicating the replacement of Br with Cl. Finally, the corresponding XRD spectra are presented in Fig. S14,† showing a shift of the characteristic peaks of CsPbBr_3_ NCs from 29.05° to 29.15° and from 38.2° to 39.2°, after chlorine treatment. On the contrary, exposure to HI gives rise to a shift of the NCs' XRD peaks to lower diffraction angles (Fig. S14†). Both of the above findings are in accordance to previous literature observations on Br–Cl anion exchange reactions in perovskite NCs.^24^,^25^,^42^,^43^ In accordance to the emission spectra, the corresponding XRD spectra remain practically unaffected upon storage of the nanocomposites for 30 days in ambient conditions (Fig. S14†).

## Conclusions

In summary, we have demonstrated a straightforward route to realize a, solid-state, anion exchange process in cesium lead halide perovskite NCs hosted into a polymer matrix. It is based on the exposure of perovskite NC:PDMS nanocomposite layers to a controlled halogen gas atmosphere. Using this method the nanocomposite absorption and emission properties can be spectrally tuned from the visible to ultraviolet, upon varying the exposure time to the respective halogen gas partial pressure. It is important to note here that the PDMS matrix constitutes a robust environment for the embedded perovskite NCs and secures their stability against humidity. The tunable optical characteristics, adjustable NC loadings and the ease of handling make the resulting nanocomposites attractive for applications in optoelectronics, *e.g.*, as color conversion materials for solid-state lighting, laser gain media, and solar light concentrators. Most importantly, all inorganic cesium lead halide perovskite NC-based nanocomposites are presented as suitable candidates for halogen gas sensing applications. Presumably, the solid-state anion exchange strategy presented here can be practically applied to other inorganic as well as organic–inorganic perovskite polymer nanocomposites.

## Funding sources

This work was supported by the State Scholarship Foundation (IKY) within the framework of the Action “Postdoctoral Researchers Support” (MIS: 5001552) from the resources of the OP “Human Resources Development, Education and Lifelong Learning” – ESPA 2014–2020 Program, contract number: 2016-050-0503-8904.

## Conflicts of interest

There are no conflicts to declare.

## Supplementary Material

Supplementary information
