# Supplementary material for: Anion exchange in inorganic perovskite nanocrystal polymer composites
Source: Chem Sci. 2018 Aug 30;9(42):8121–6. doi: 10.1039/c8sc02830c (PMC6238712; doi:10.1039/c8sc02830c)
Supplement: Supplementary file 1 [file SC-009-C8SC02830C-s001.pdf]

Electronic Supplementary Material (ESI) for Chemical Science.
This journal is © The Royal Society of Chemistry 2018

Electronic Supplementary Information

Anion Exchange in Inorganic Perovskite Nanocrystal Polymer Composites

Maria Sygletou,*[a] Maria-Eleni Kyriazi,[b] Antonios G. Kanaras[b] and Emmanuel Stratakis *[ac]

[a]Institute of Electronic Structure and Laser, Foundation for Research and Technology - Hellas, Heraklion, 71110, Crete, Greece
[b]Physics and Astronomy, Faculty of Physical Sciences and Engineering, University of Southampton, Southampton, SO171BJ, UK
[c]Department of Materials Science and Technology, University of Crete, Heraklion 71003, Crete, Greece
* Corresponding authors: masyg@iesl.forth.gr, stratak@iesl.forth.gr

Synthesis Methods

Cubic shaped, $CsPbBr_3$ NCs with an average size of ~10 nm were prepared following a modified procedure that was first reported by Protesescu *et al.*[1](Fig. S1).

Cs-oleate preparation: Caesium acetate (0.884 g, Sigma Aldrich, 99.9 %) was transferred to a 100 mL 3-neck flask along with 1-octadecene (40 mL, Sigma Aldrich, 90 %) and oleic acid (2.5 mL, Sigma Aldrich, 90 %). This was heated under vacuum for 1 h at 120 °C and then placed under Ar atmosphere.

Synthesis of $CsPbBr_3$ NCs: 1-octadecene (5 mL), $PbBr_2$ (0.069 g, Sigma Aldrich, 98 %), oleylamine (0.5 mL, Fisher Scientific, 80-90 %) and oleic acid (0.5 mL) were added to a 25 mL 3-neck flask. The solution was heated at 120 °C for 1 h under vacuum making sure that complete solubilisation of the $PbBr_2$ salt had taken place. After switching to Ar atmosphere and raising the temperature to 170 °C the Cs-oleate solution (0.4 mL, prepared as described above) was quickly injected into the solution and the reaction mixture was cooled in an ice-water bath. UV-Vis absorption and PL spectra were recorded in order to evaluate the optical properties of the NCs (Fig. S2).

Preparation of nanocomposites and anion exchange processes

Preparation of polymer:NC composites:
Sylgard 184 silicone elastomer was supplied as a two-part liquid component kit, a pre-polymer base (part A) and a crosslinking curing agent (part B)[31]. The polymer's stiffness was controlled by varying the crosslinking density. In our study, a ratio of 1:10 (weight ratio, w/w curing agent to prepolymer), was used. In a first step, the perovskite NC solution was added to the mixture of the polymer together with the curing agent, in various w/w ratios (Sylgard 184 silicone elastomer to NCs solution; 2:1, and 4:1). Following the preparation of the mixture, the solution was drop casted on quartz substrates to create thin films. For thicker layers, the solution was placed into teflon molds (x=1.3 cm, y=1.3 cm, z=0.2 cm). In order to fabricate bubble free test samples the layers were thoroughly degassed in a vacuum desiccator at low pressure overnight at 70 °C. The resulting thickness of the films on the quartz substrates was approximately 0.5 mm and for the samples on Teflon substrates the thickness was 2 mm (Figure 2, inset). Indicative SEM images of the samples' surfaces are shown in Figure S3.

Solid-state chlorination process:
Following their preparation, the samples were placed inside a vacuum chamber. Cl_2 gas was controllably (up to 70 mbar partial pressure) inserted inside the chamber and the samples were exposed to this chlorine environment for various time intervals. The amount of Cl_2 gas inserted into the chamber was controlled *via* a pressure gauge. After reaction with chlorine, the perovskite samples were held at reduced pressure for 3 h to remove surface-adsorbed chlorine before spectroscopy analysis.

Solid-state iodination process:
For the iodination of the $CsPbBr_3$ NCs and the chlorinated $CsPbBr_3$ NCs hosted in polymer matrix, the samples were placed in a quartz tube containing 0.25 g of iodine (I_2) crystals. The films were placed on the top of the tube facing the iodine crystals in order to avoid contact between solids. The tube was heated at 100 ºC for 10 minutes. After reaction with iodine, the perovskite samples were held at reduced pressure for 3 h to remove surface-adsorbed iodine before spectroscopy analysis.

Characterization Methods:

Spectroscopic Characterization:

Optical Absorption Spectroscopy (UV-vis): UV-Vis absorption spectra of the NC colloids and NCs:PDMS composites were collected at room temperature using a Perkin Elmer, LAMBDA 950 UV/VIS/NIR spectrophotometer. The colloidal solutions were placed in quartz cuvettes of 1 mm thickness without further dilution.

Laser Induced Fluorescence Spectroscopy (LIF): The colloidal NC solutions were placed in quartz cuvettes of 1 mm thickness, while the polymer/perovskites nanocomposite layers were fixed on a X-Y stage. Then, the respective fluorescence spectra were recorded at room temperature. For the samples' excitation, a KrF excimer nanosecond laser, operating at 248 nm wavelength was utilized. The pulse duration was 20 ns, the excitation energy was 0.5 mJ and the laser beam had a diameter of 3.6 mm (corresponding to a fluence of 5 mJ/cm^2). The fluorescence spectra were recorded by a PTI Technology monochromator which was connected to an Andor iStar 734 Series, time resolved, cooled and Intensified Charge Coupled Device (ICCD). The fluorescence signal of the samples was collected and guided to the spectrograph by an optical fiber.

Fourier Transform Infra-Red Spectroscopy (FTIR): FT-IR (transmission) experiments were carried out with a Bruker Vertex 70v FT-IR vacuum spectrometer, in a spectral range of 7500 – 350 cm^{-1}. A broad band KBr detector and a room temperature broad band triglycine sulfate (DTGS) detector were used, while interferograms were collected at a 4 cm^{-1} resolution (8 scans), analysed with a Blackman-Harris function, and Fourier transformed with two levels of zero filling to yield spectra encoded at 2 cm^{-1} intervals. Before scanning the samples, an empty holder (in vacuum) background measurement was recorded.

X-ray Photoelectron Spectroscopy (XPS): Surface analysis studies were performed in a UHV chamber (P<10-9 mbar) equipped with a SPECS LHS-10 hemispherical electron analyzer and a dual anode Mg/Al x-ray gun. The XPS measurements were carried out at room temperature using unmonochromatized AlKa with a pass energy of 97 eV giving a full width at half maximum (FWHM) of 1.9 eV for the Au 4f7/2 peak. The analyzed area was a rectangle with dimensions of 2.5x4.5mm^2. The XPS core level spectra were analyzed using a fitting routine, which can decompose each spectrum into individual mixed Gaussian-Lorentzian peaks after a Shirley background subtraction.

Structural Characterization:

Scanning Electron Microscopy (SEM): SEM served to investigate the morphology of the samples before and after interaction with Cl_2. To that purpose the samples were coated by platinum using sputtering and analyzed by a JEOL JSM-6390LV system.

Transmission Electron Microscopy (TEM): TEM images were taken using a Hitachi H7000 TEM with a bias voltage of75 kV.

X-Ray Diffraction Analysis (XRD): XRD studies were performed on a Rigaku D/MAX-2000H rotating anode diffractometer with Cu Kα radiation, equipped with a secondary graphite monochromator. The XRD data were collected at room temperature over a 2θ scattering range of 10–40°, with a step of 0.05° and a counting time of 20 s/step. The all inorganic cesium lead halide perovskites:PDMS nanocomposites were placed onto a glass sample holder.

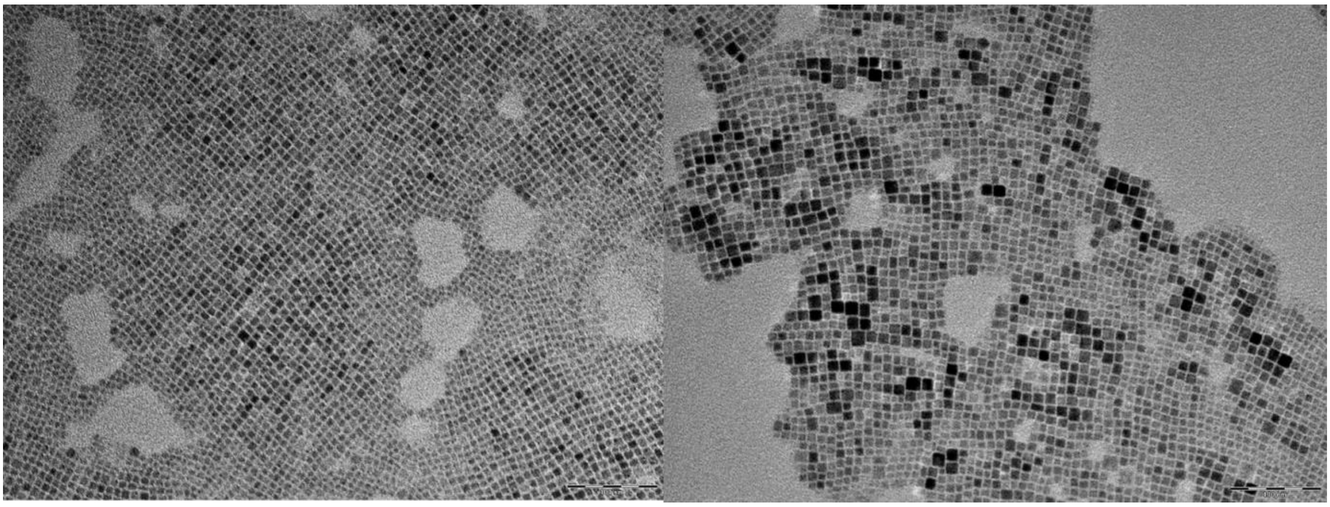

Fig. S1 TEM images of $CsPbBr_3$ NCs in hexane, at different magnifications. Scale bars are 100 nm.

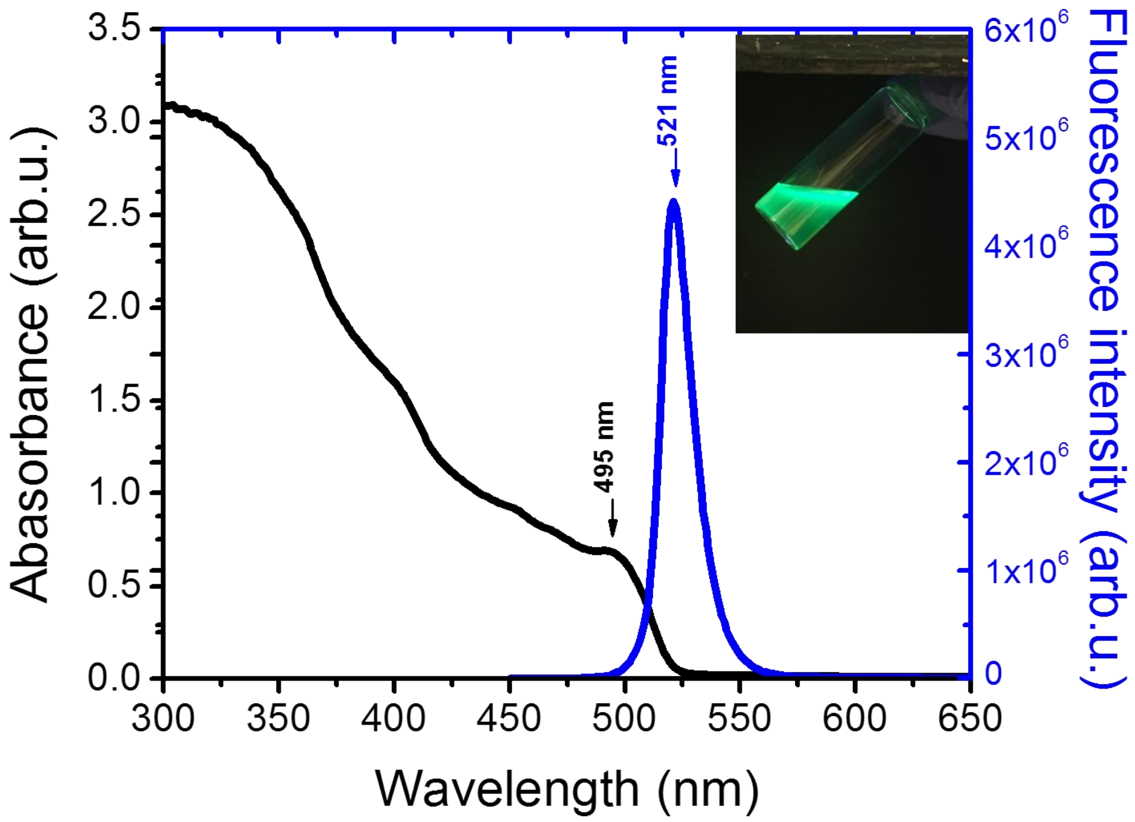

Fig. S2 UV-Vis absorption and fluorescence spectra of $CsPbBr_3$ NCs, in hexane. The inset shows $CsPbBr_3$ NCs in hexane, under UV light.

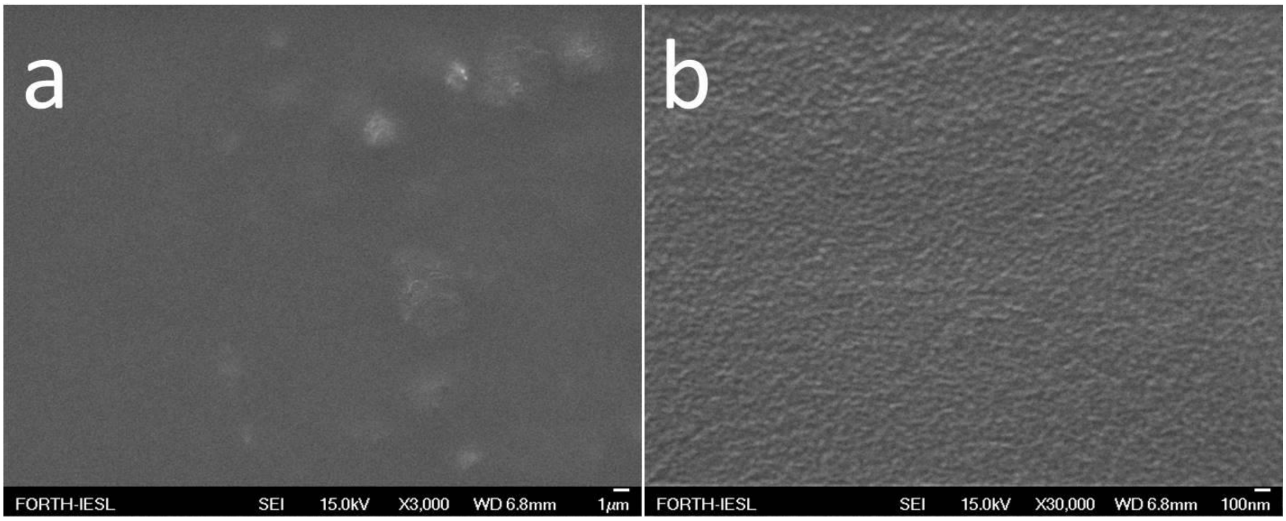

Fig. S3 SEM images of $CsPbBr_3$/PDMS nanocomposites at different magnifications.

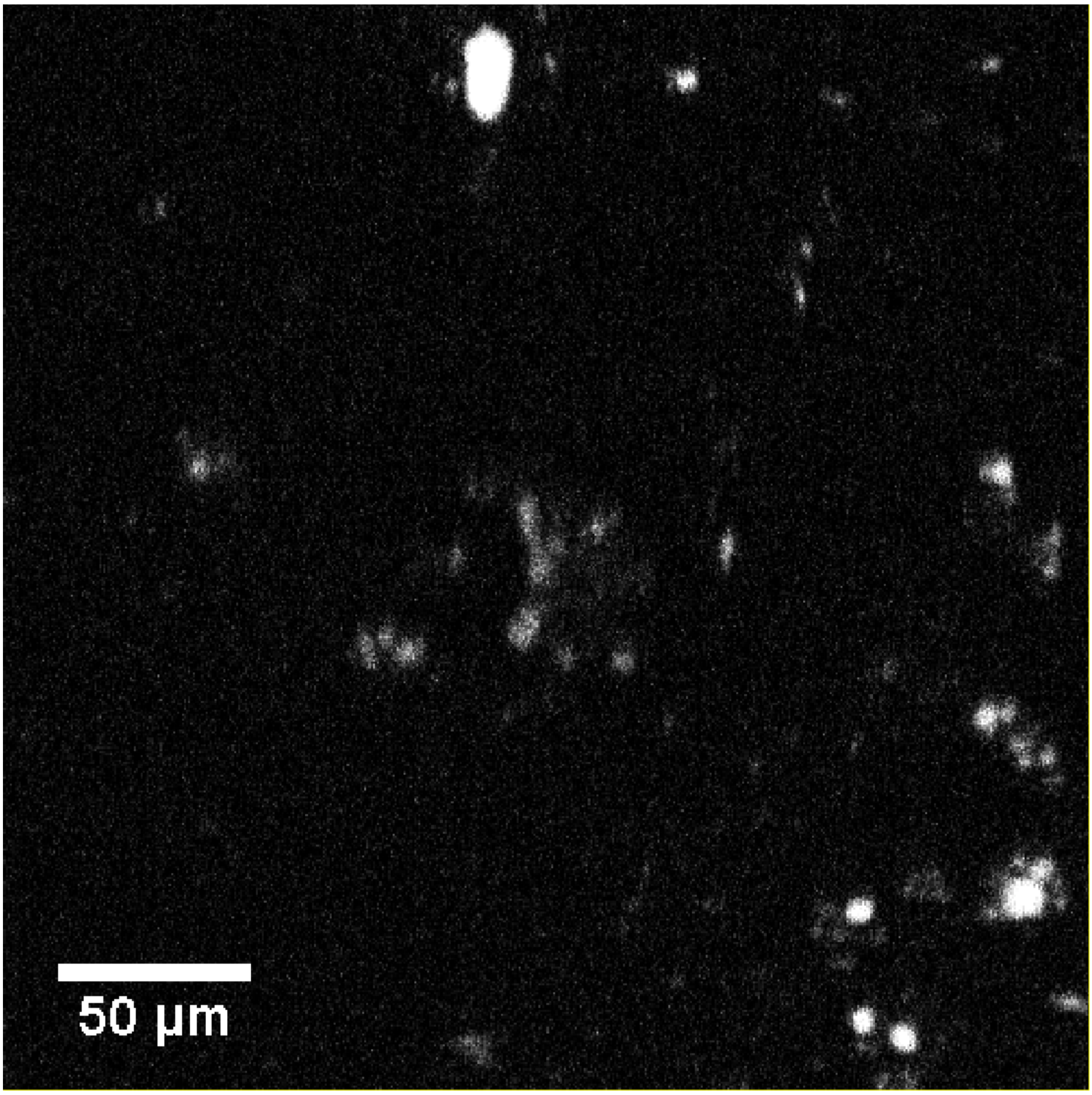

Fig. S4 Two-Photon Excited Fluorescence (TPEF) Microscopy image of $CsPbBr_3$/PDMS nanocomposites (thickness= 2mm). The white spots are the $CsPbBr_3$ NCs while the larger spots are clusters of the NCs.

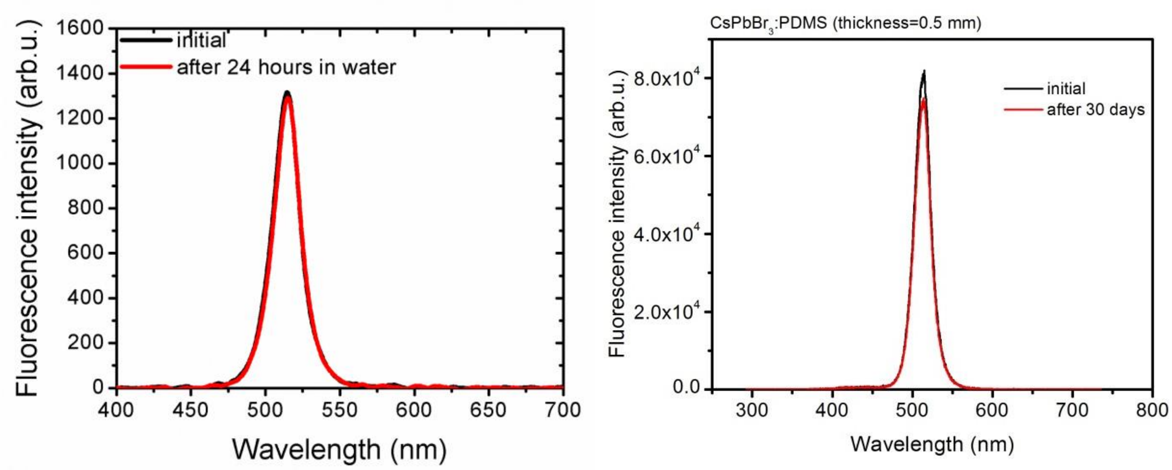

Fig. S5 (Left) Fluorescence spectra of $CsPbBr_3$/PDMS nanocomposites before and after 24 h in water (black line: initial spectrum, red line: spectrum acquired after 24 h in water); (Right) . Fluorescence spectra of $CsPbBr_3$:PDMS nanocomposite before (black line) and 30 days after (red line) chlorine treatment, upon storage in ambient conditions.

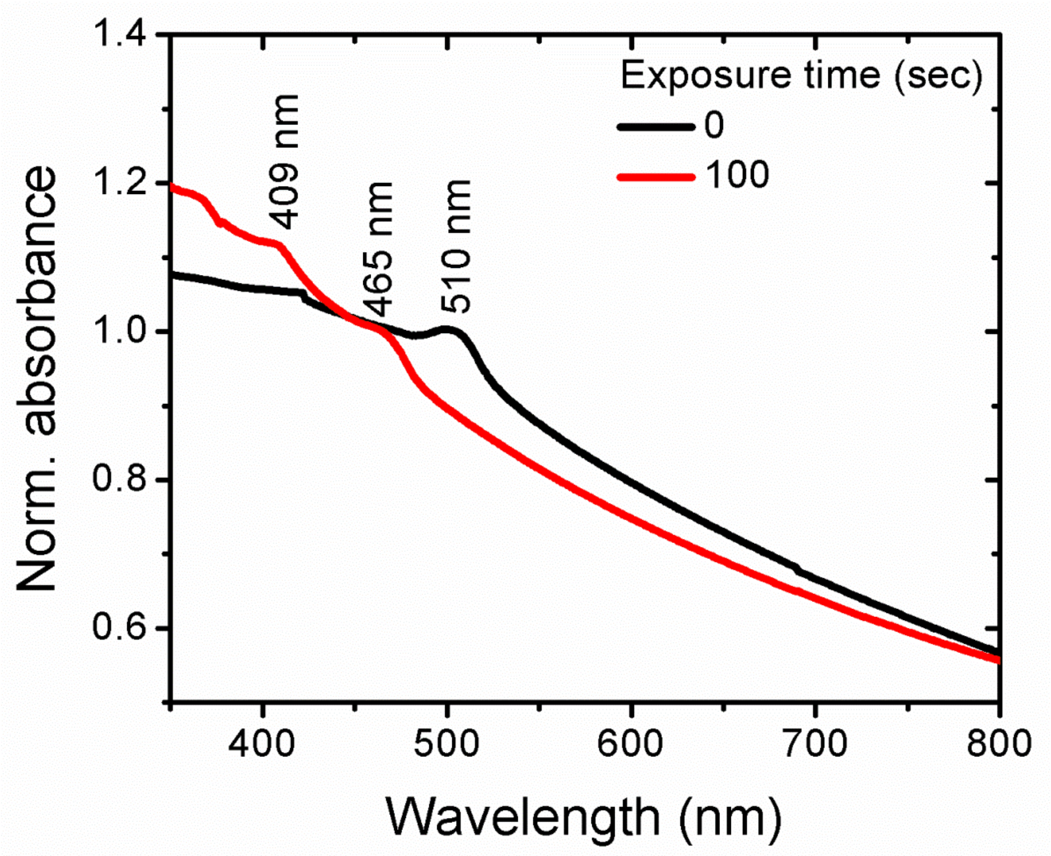

Fig. S6 Normalized UV-Vis absorption spectra of NCs/PDMS nanocomposites of a 2 mm thickness, before and after exposure to Cl_2 gas for 100 s.

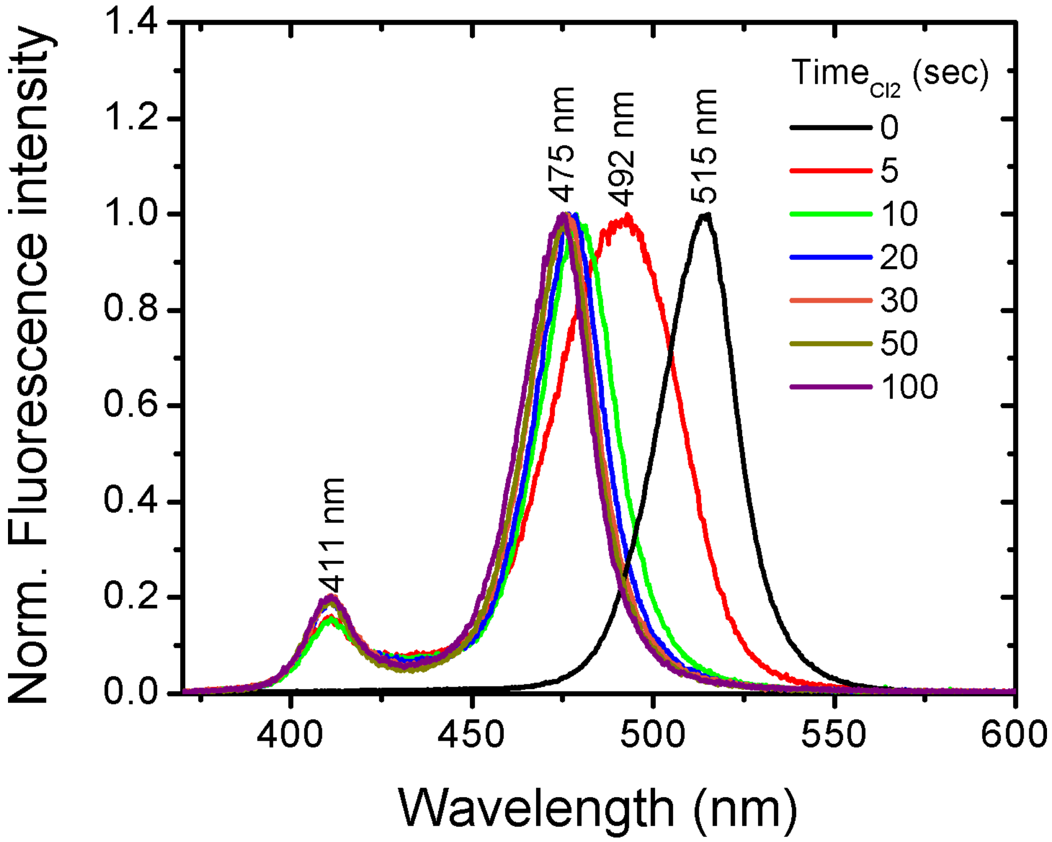

Fig. S7 a) Normalized fluorescence spectra of NCs/PDMS nanocomposites of a 2 mm thickness, before and after exposure to Cl_2 gas for various time intervals.

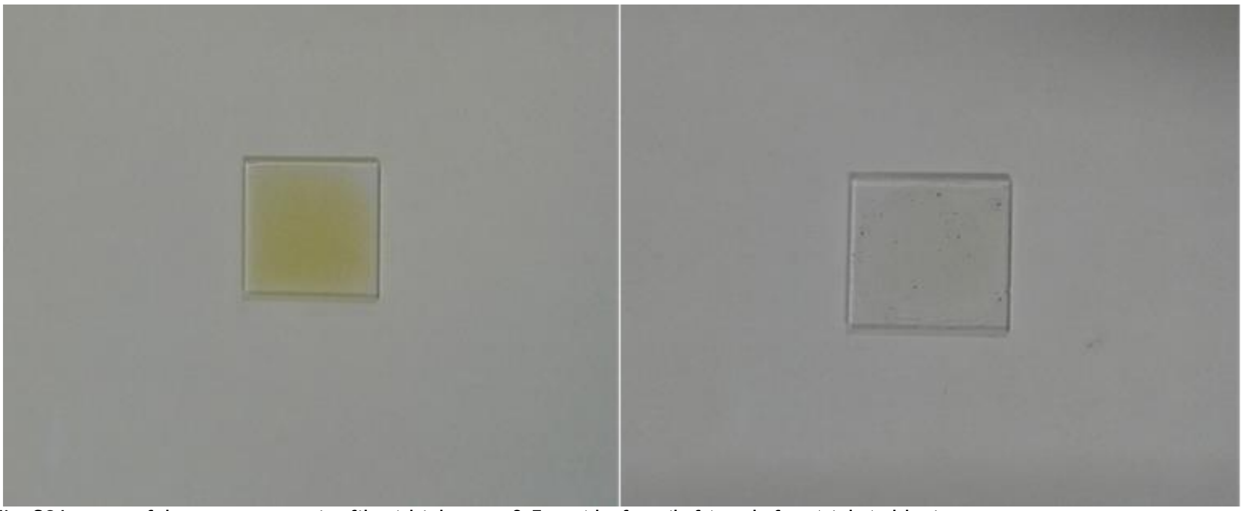

Fig. S8 Images of the nanocomposite film (thickness = 0.5 mm) before (left) and after (right) chlorine treatment for 100 s.

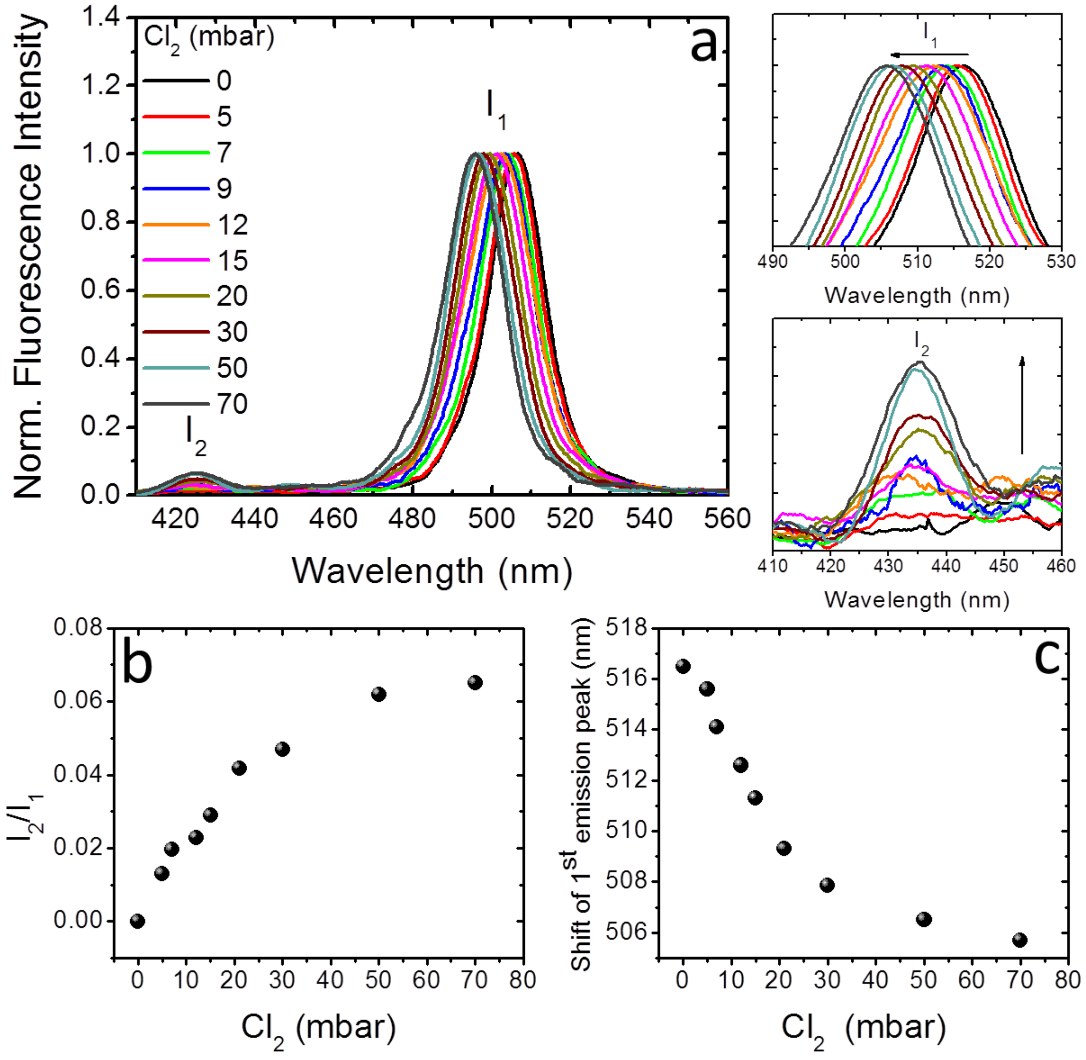

Fig. S9 a) Normalized fluorescence spectra of $CsPbBr_3$/PDMS nanocomposites (thickness = 2 mm) at different Cl_2 pressure values. (Right) Evolution of the fluorescence signal of the initial peak (top) and of the peak that appears after chlorine treatment (bottom). b) Fluorescence intensity ratio of the two emission peaks and c) wavelength shift of the first emission peak of the nanocomposite, as a function of the pressure of Cl_2 gas.

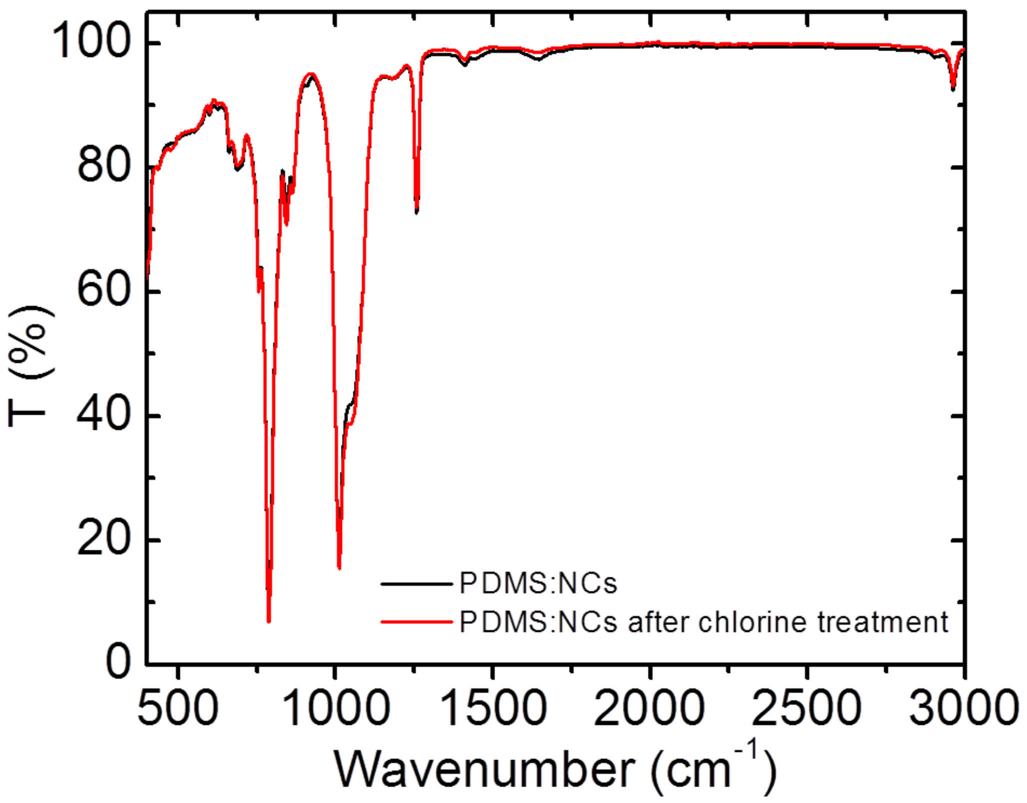

Fig. S10 FTIR-ATR spectra of $CsPbBr_3$ NC:PDMS layers prior (black line) and after (red line) chlorine treatment for 10 minutes.

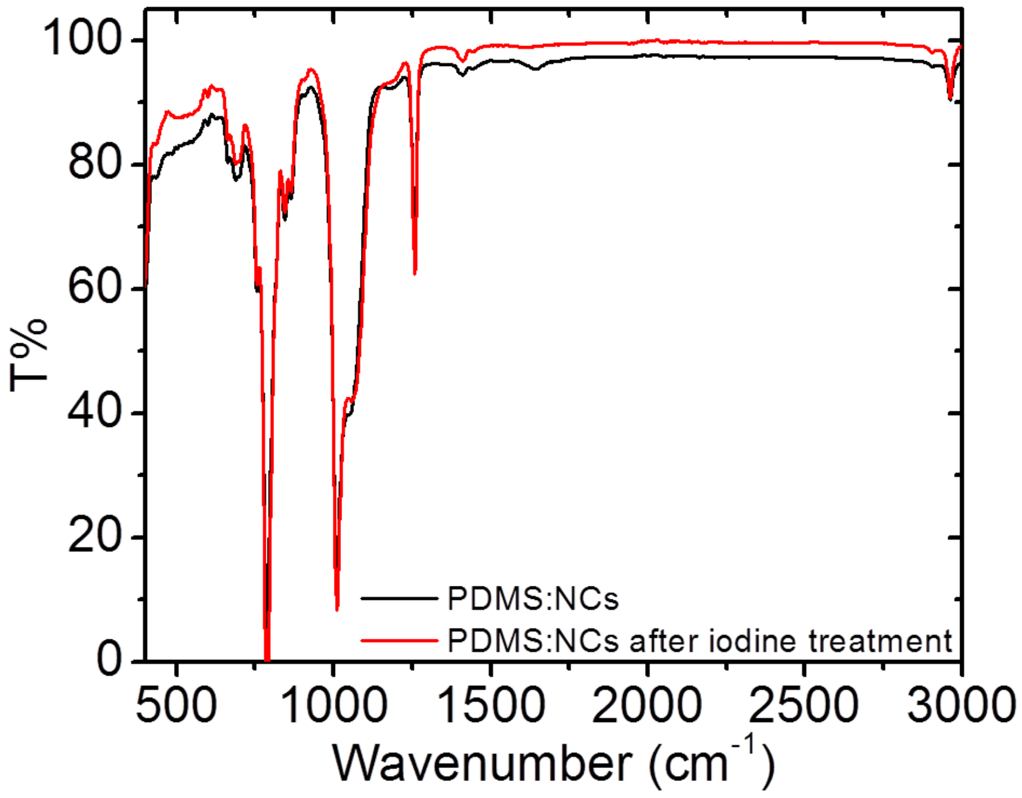

Fig. S11. FTIR-ATR spectra of $CsPbBr_3$ NC:PDMS layers prior (black line) and after (red line) iodine treatment under ambient conditions for 10 minutes.

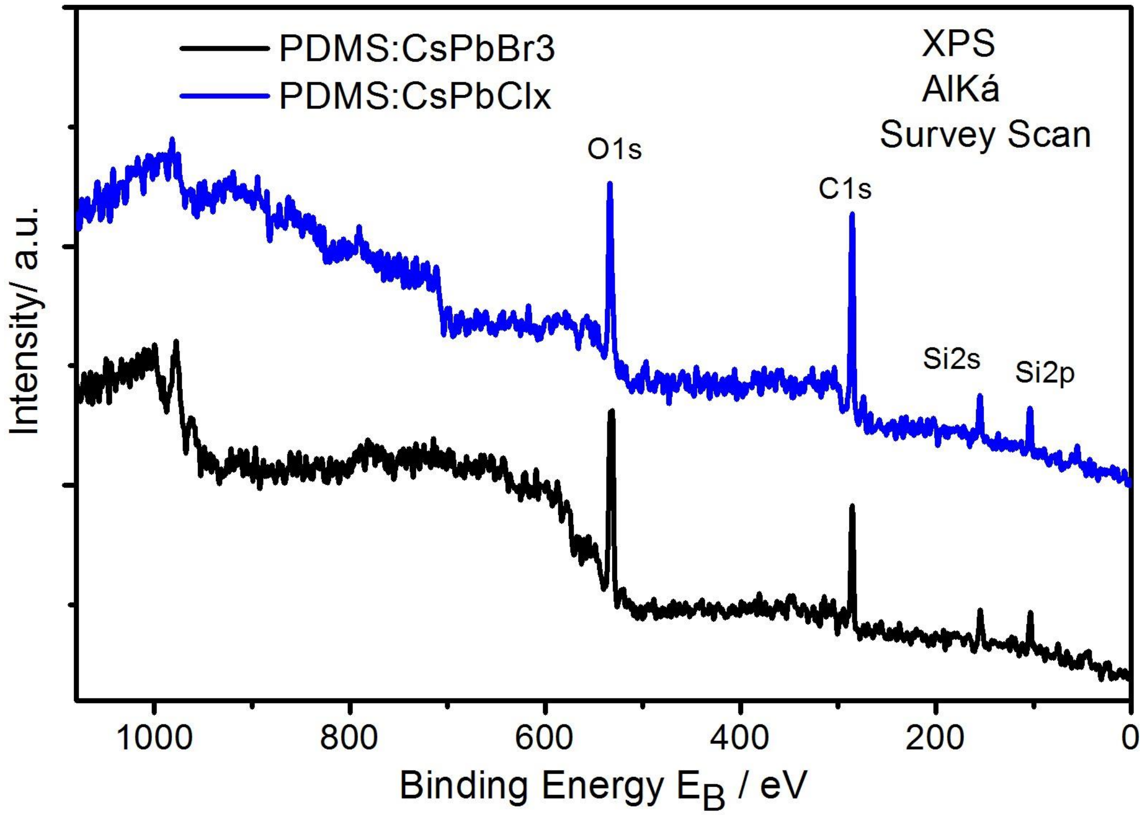

Fig. S12. XPS survey scans of $CsPbBr_3$:PDMS before (black line) and after (blue line) exposure to Cl_2 gas (70 mbar) for 10 minutes.

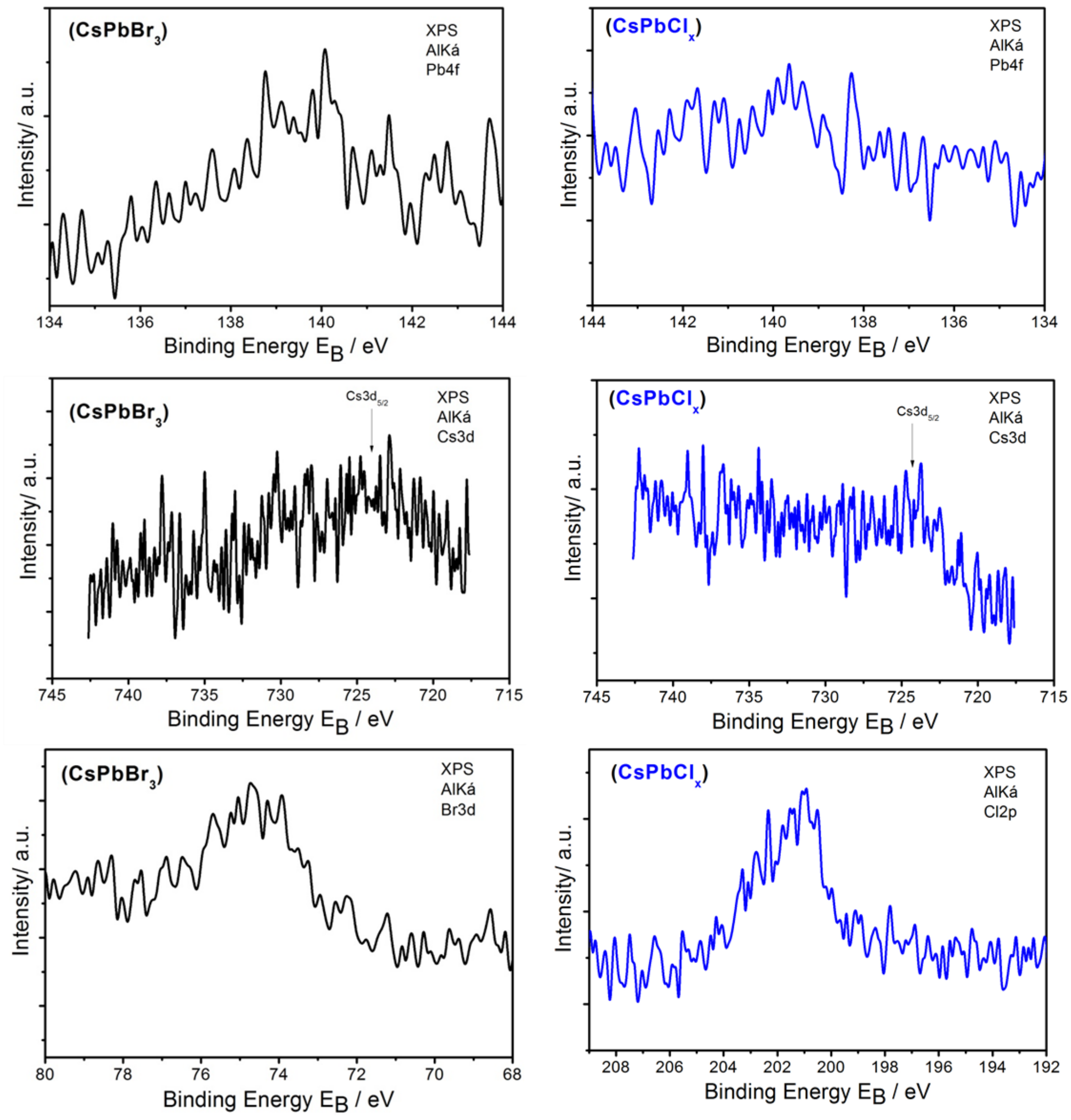

Fig. S13. XPS spectra of $CsPbBr_3$:PDMS before (black line) and after (blue line) exposure to Cl_2 gas (70 mbar) for 10 minutes.

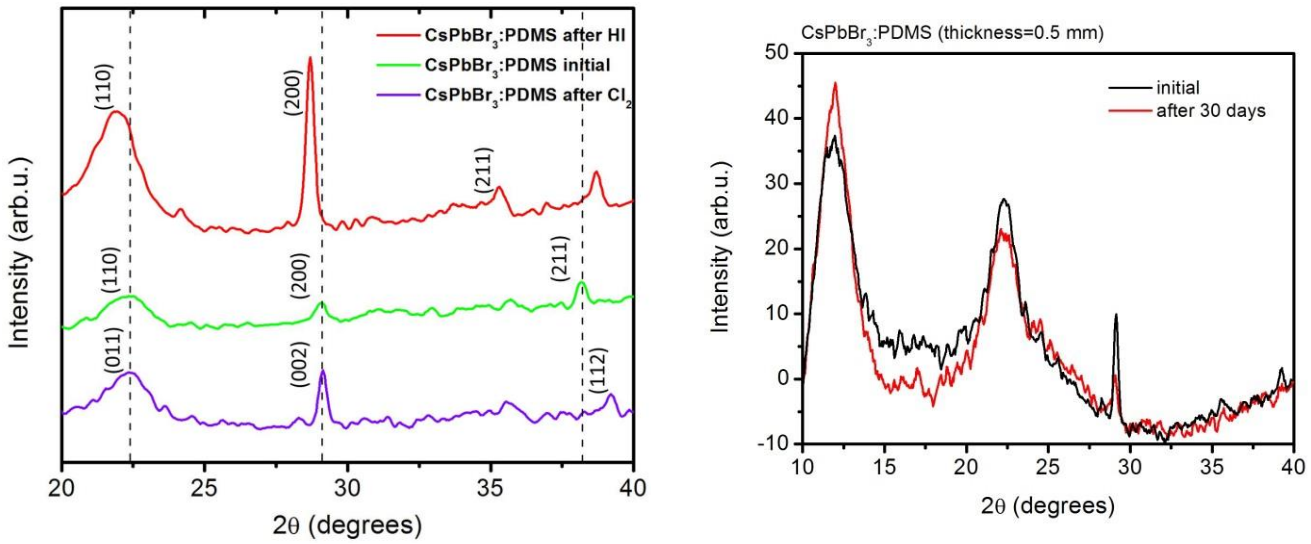

Fig. S14. XRD patterns of initial CsPbBr$_3$:PDMS nanocomposite (green line), after chlorine treatment (blue line) and after HI treatment (red line)..b) XRD patterns of CsPbBr$_3$:PDMS of CsPbBr$_3$:PDMS nanocomposite before (black line) and 30 days after chlorine treatment (red line), upon storage in ambient conditions.

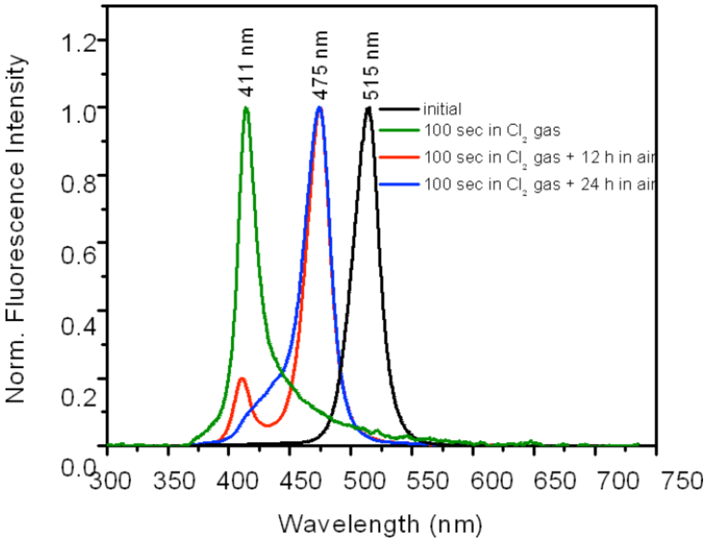

Fig. S15 Normalized fluorescence spectra of the pristine CsPbBr$_3$:PDMS nanocomposites of thickness 0.5 mm (black line), immediately after chlorine treatment for 100 seconds (green line line), 12h after chlorine treatment (red line) and 24 hours after chlorine treatment (blue line).

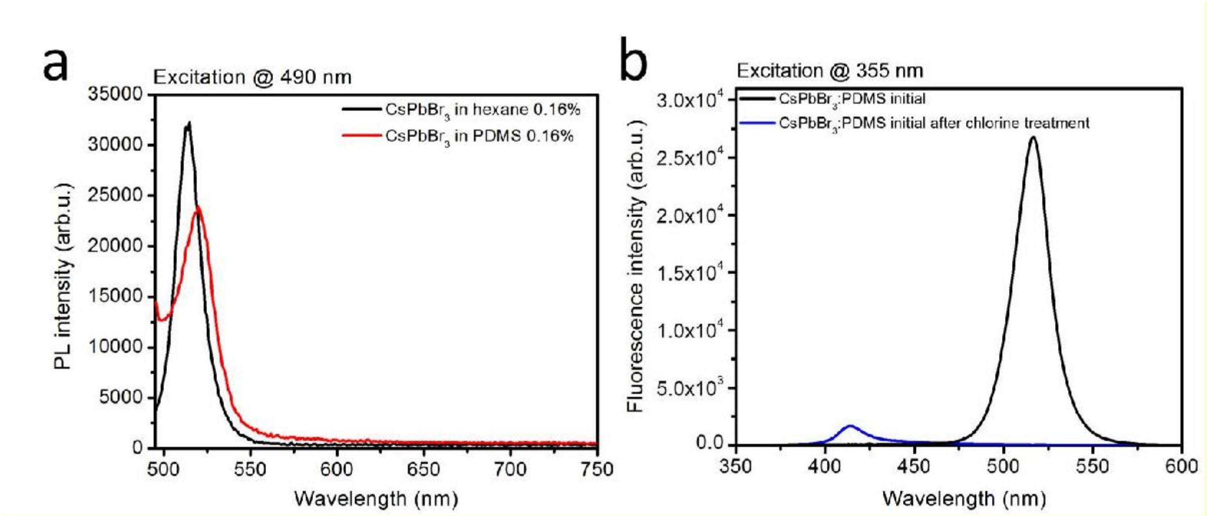

Fig. S16. a) Fluorescence spectra of $CsPbBr_3$ NCs in hexane (black line) and $CsPbBr_3$ in PDMS nanocomposite (red line) following excitation at 490 nm which is the first exciton peak position. b) Fluorescence spectra of $CsPbBr_3$:PDMS nanocomposite of thickness 0.5 mm before (black line) and after (blue line) chlorine treatment, following excitation at 355 nm.

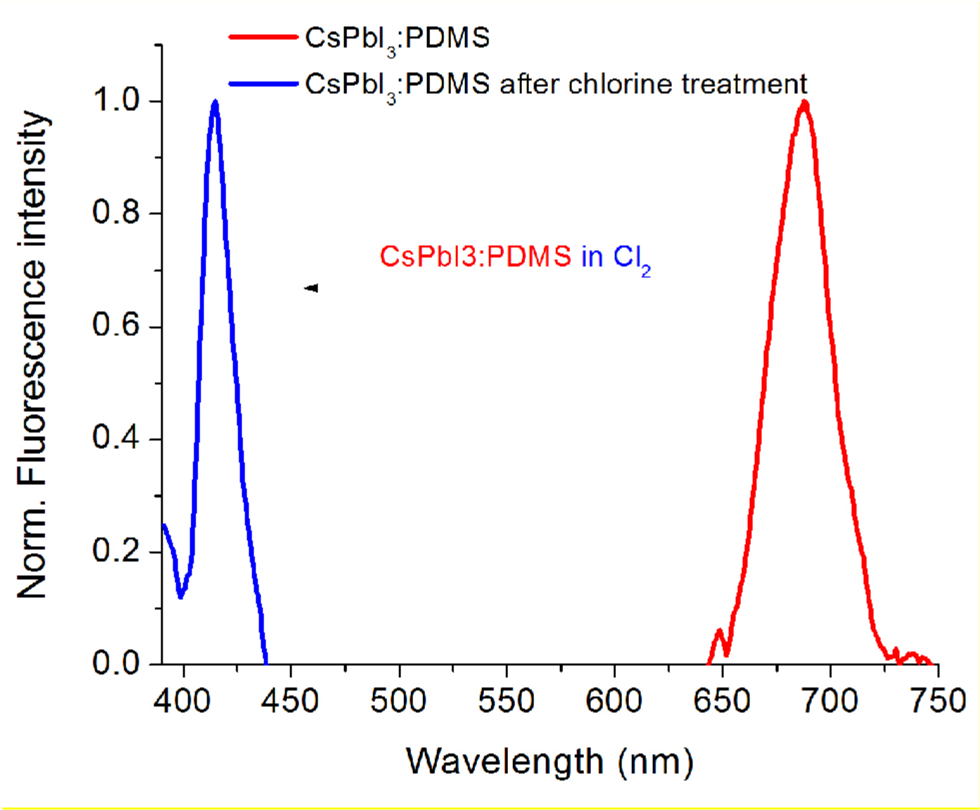

Fig. S17 Fluorescence spectra of PDMS:$CsPbI_3$ NCs nanocomposite layers before (red line) and after chlorine treatment (blue line).

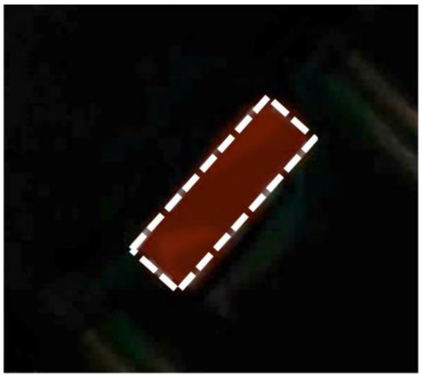

Fig. S18. Image of $CsPbI_3$:PDMS nanocomposite of thickness 0.5 mm under UV excitation.
